# Exploring Extended Warheads toward Developing Cysteine-Targeted Covalent Kinase Inhibitors

**DOI:** 10.1021/acs.jcim.4c00890

**Published:** 2024-12-10

**Authors:** Zheng Zhao, Philip E. Bourne

**Affiliations:** School of Data Science and Department of Biomedical Engineering, University of Virginia, Charlottesville, Virginia 22904, United States

## Abstract

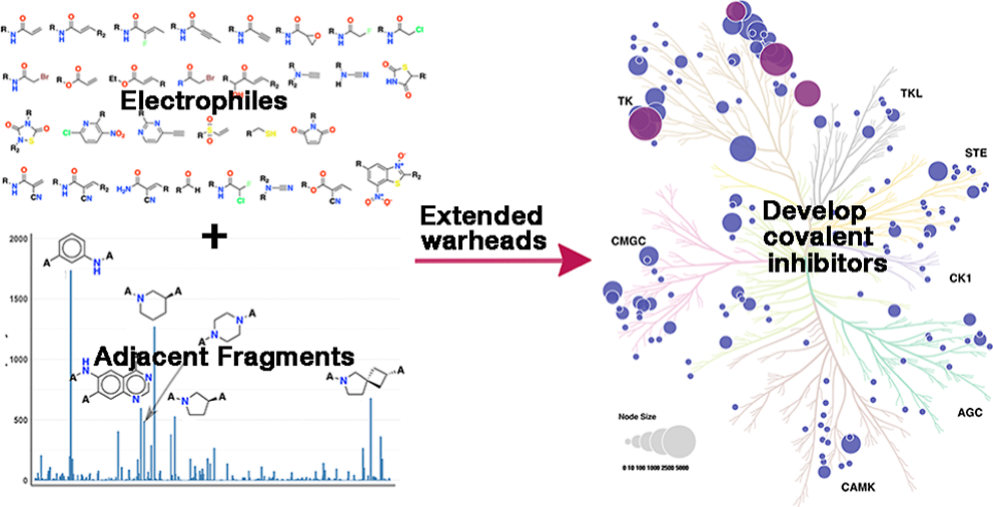

In designing covalent kinase inhibitors (CKIs), the inclusion of electrophiles as attacking warheads demands careful choreography, ensuring not only their presence on the scaffold moiety but also their precise interaction with nucleophiles in the binding sites. Given the limited number of known electrophiles, exploring adjacent chemical space to broaden the palette of available electrophiles capable of covalent inhibition is desirable. Here, we systematically analyze the characteristics of warheads and the corresponding adjacent fragments for use in CKI design. We first collect all the released cysteine-targeted CKIs from multiple databases and create one CKI data set containing 16,961 kinase-inhibitor data points from 12,381 unique CKIs covering 146 kinases with accessible cysteines in their binding pockets. Then, we analyze this data set, focusing on the extended warheads (i.e., warheads + adjacent fragments)—including 30 common warheads and 1344 unique adjacent fragments. In so doing, we provide structural insights and delineate chemical properties and patterns in these extended warheads. Notably, we highlight the popular patterns observed within reversible CKIs for the popular warheads cyanoacrylamide and aldehyde. This study provides medicinal chemists with novel insights into extended warheads and a comprehensive source of adjacent fragments, thus guiding the design, synthesis, and optimization of CKIs.

## 1 Introduction

As of November 29, 2023, 80 kinase-targeted small molecule drugs have been approved by the United States Food and Drug Administration (FDA), illustrating the value of kinase drug development for the treatment of a variety of diseases,^1−3^ such as nonsmall cell lung cancer.^4,5^ However, these drugs target just 24 kinases, a small part of the human kinome, implying considerable scope remains for developing kinase inhibitors, probes, and drugs.^6−8^

**Figure 1:**
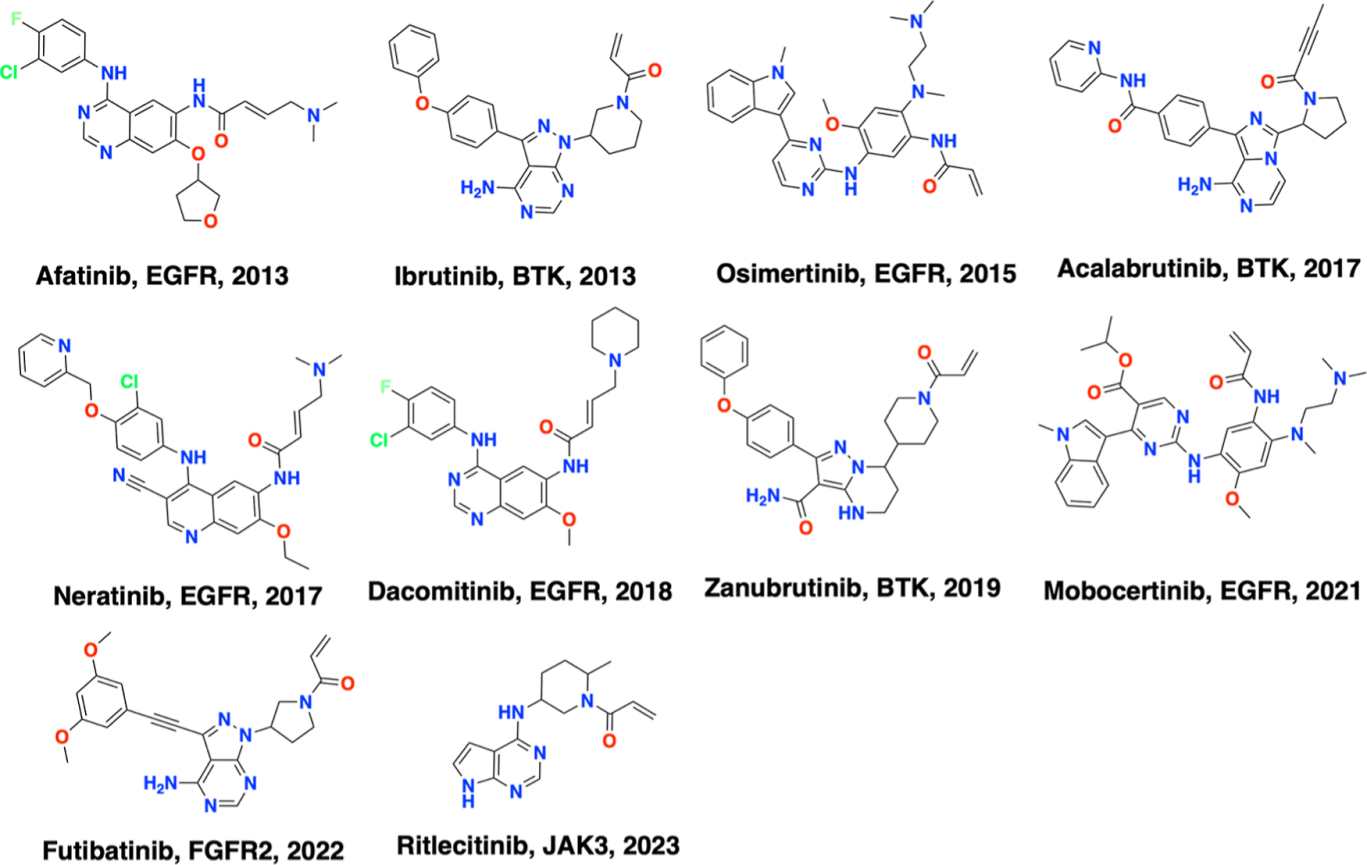
Ten FDA-approved CKIs showing their primary target and year of approval.

Kinase inhibitors published so far can be divided into Type-I, -II, -III, and Type IV according to their corresponding binding modes.^9,10^ Type-I inhibitors bind into the ATP binding site with the active (DFG-in) kinase conformation like ATP.^2^ Typically Type-I inhibitors are composed of an adenine-analog core fragment that forms 2–3 hydrogen-bond interactions with the hinge region.^11,12^ Type-II inhibitors not only bind into the ATP binding site with the inactive (DFG-out) kinase conformations but also extend into the nearby allosteric pocket, where Type-III inhibitors interact.^13,14^ In contrast, Type-IV allosteric inhibitors bind to pockets away from the ATP binding site, such as those pockets distributed on the C-terminal lobe.^14^ To achieve favorable selectivity and potency, other types of inhibitors with promising features have been developed, such as macrocyclic inhibitors and covalent inhibitors.^15−18^ Macrocyclic inhibitors refer to, as the name suggests, inhibitors possessing a macrocycle (>12-membered ring).^19^ Covalent inhibitors are those compounds that bind into the binding pockets covalently reacting with noncatalytic nucleophilic residues.^20,21^ Due to noncatalytic nucleophilic residues being poorly conserved, covalent interactions offer more opportunities to enhance selectivity across the whole kinome. Thus far, ten covalent kinase drugs have been approved by the FDA, targeting epidermal growth factor receptor (EGFR), Bruton’s tyrosine kinase (BTK), fibroblast growth factor receptor 2 (FGFR2), and Janus kinase 3 (JAK3), respectively (Figure 1).^5^ Since designing covalent inhibitors by manipulating the electrophiles is attractive, tens of common electrophiles used as warheads (e.g., acrylamide) have been applied to the discovery of covalent kinase inhibitors (CKIs), with cysteine, tyrosine, and threonine as nucleophiles.^22−26^ However, such a small number of electrophiles is limiting; thus, it is helpful to extend the design of warheads to include adjacent fragments. Here, we define adjacent fragments as those fragments that are immediately adjacent to the warhead and connect the warhead and scaffold moieties. Combining adjacent fragments and warheads offers more combinations to achieve binding.

Fortunately, tens of thousands of CKIs have been published and cumulatively provide a basis to explore adjacent fragments.^23,27^ We focus on cysteine-targeted CKIs since they are widely used and have available a variety of bioactive data.^23^ We first integrate multiple kinase data sources into a curated CKI data set and investigate CKI chemical space, structural characteristics, and target space with emphasis on the features of extended warheads, with emphasis on reversible CKIs (RCKIs).

## 2 Results

### 2.1 CKI Data Set

**Figure 2:**
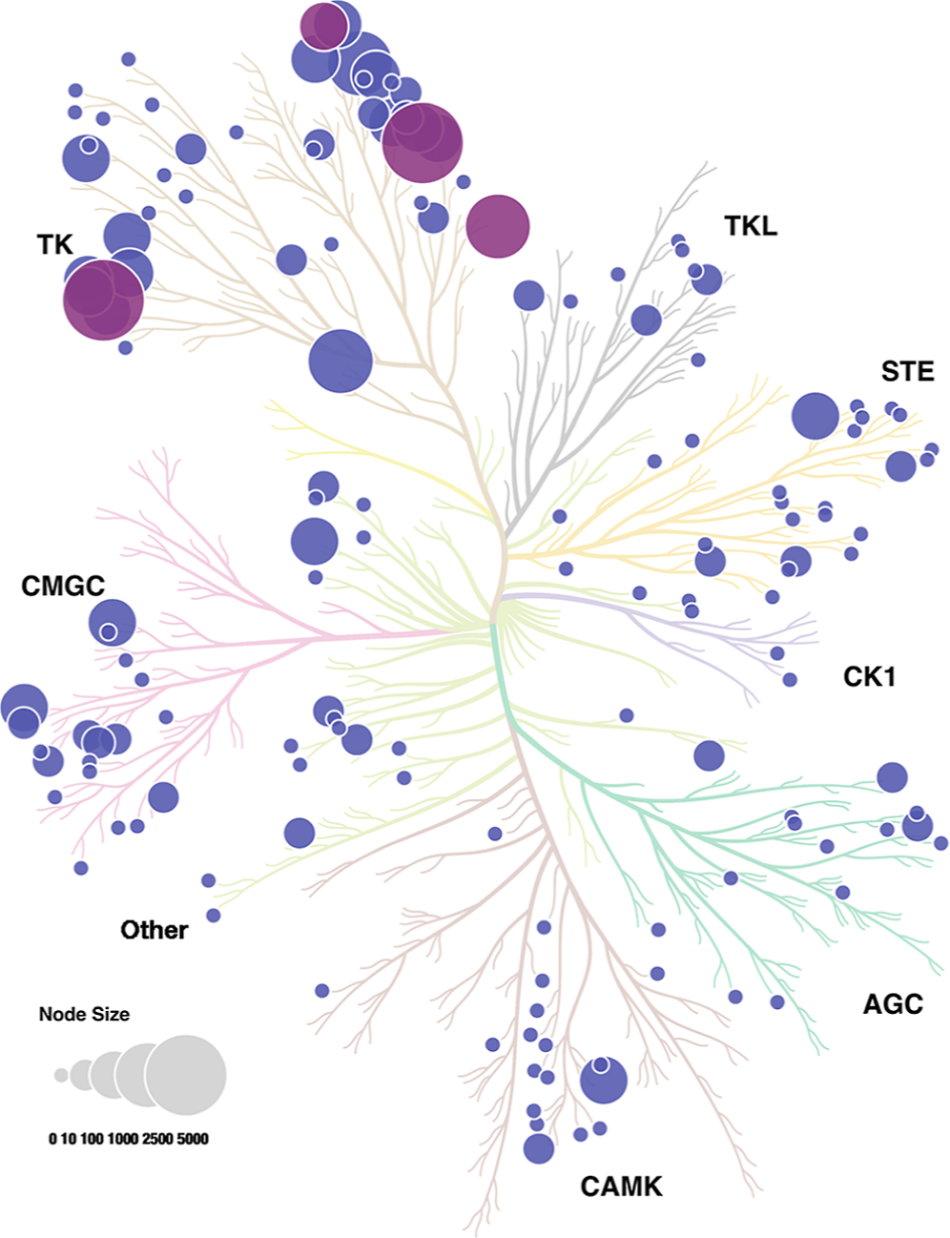
Distribution of CKIs on the human kinome phylogenetic tree. Each point represents one kinase with released (violet), or approved CKIs (purple). The point size is proportional to the number of CKIs.

**Figure 3:**
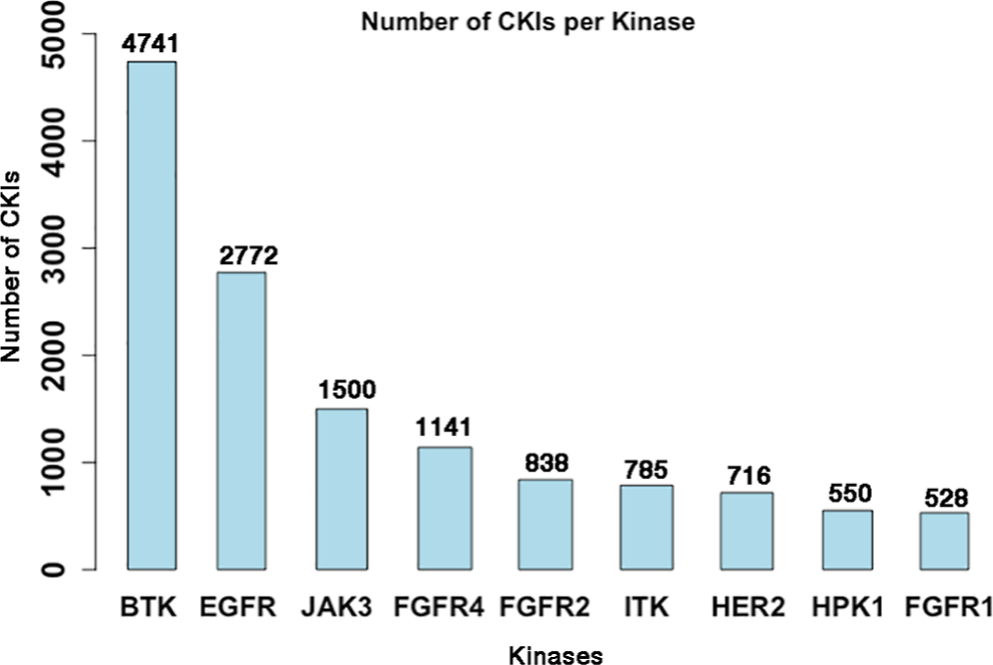
Top 9 kinases with >500 CKIs.

We integrated all CKIs from the BindingDB and CheMBL databases and an in-house CKI data set (see the Method section). After curation (see the Method section), we obtained 16,961 electrophiles-equipped bioactive data points including 12,381 unique CKIs, for 146 out of 208 kinases that possess available cysteines near the ATP binding site.^2^Figure 2 shows the human kinome phylogenetic tree with the distributions of the 146 kinases, which cover all protein kinase groups (TK, TKL, STE, CK1, AGC, CAMK, CMGC, and other).^1,28^ 95 out of 146 kinases have single-digit curated CKIs while the top 9 kinases have more than 500 CKIs (Figure 3). Interestingly, the 10 approved CKIs (Figure 1) are all distributed among the top 9 kinases (BTK, EGFR, FGFR2, and JAK3). This indicates that CKI’s discovery is challenging, since although thousands of CKIs have been published or patented, only very few ultimately enter the clinic. Conversely, this distribution implies that more promising covalent drugs targeting more kinases are possible with additional CKIs.

### 2.2 CKI Warheads

**Figure 4:**
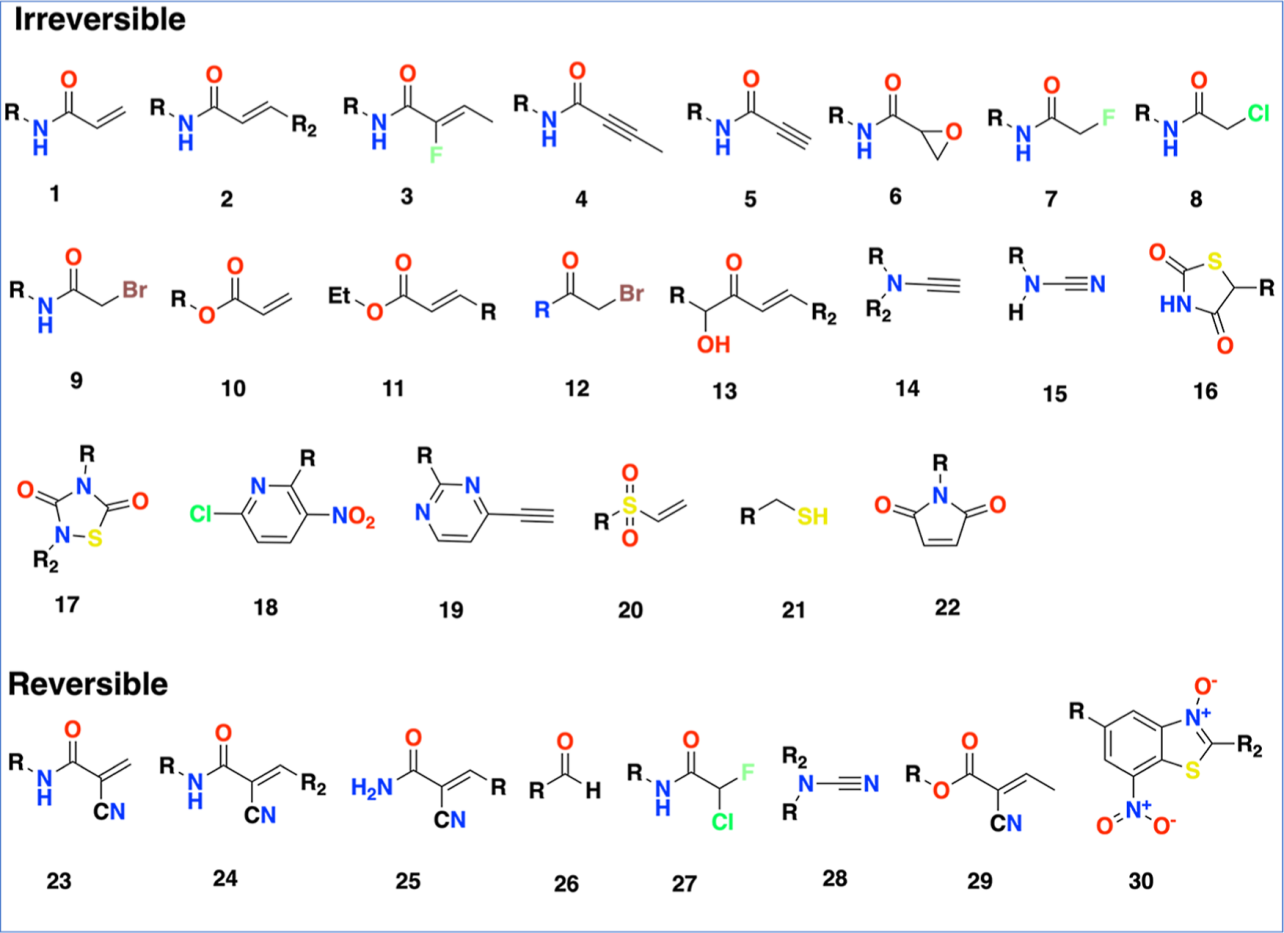
CKI warheads **1**–**22** are irreversible and **23**–**30** are reversible.

**Figure 5:**
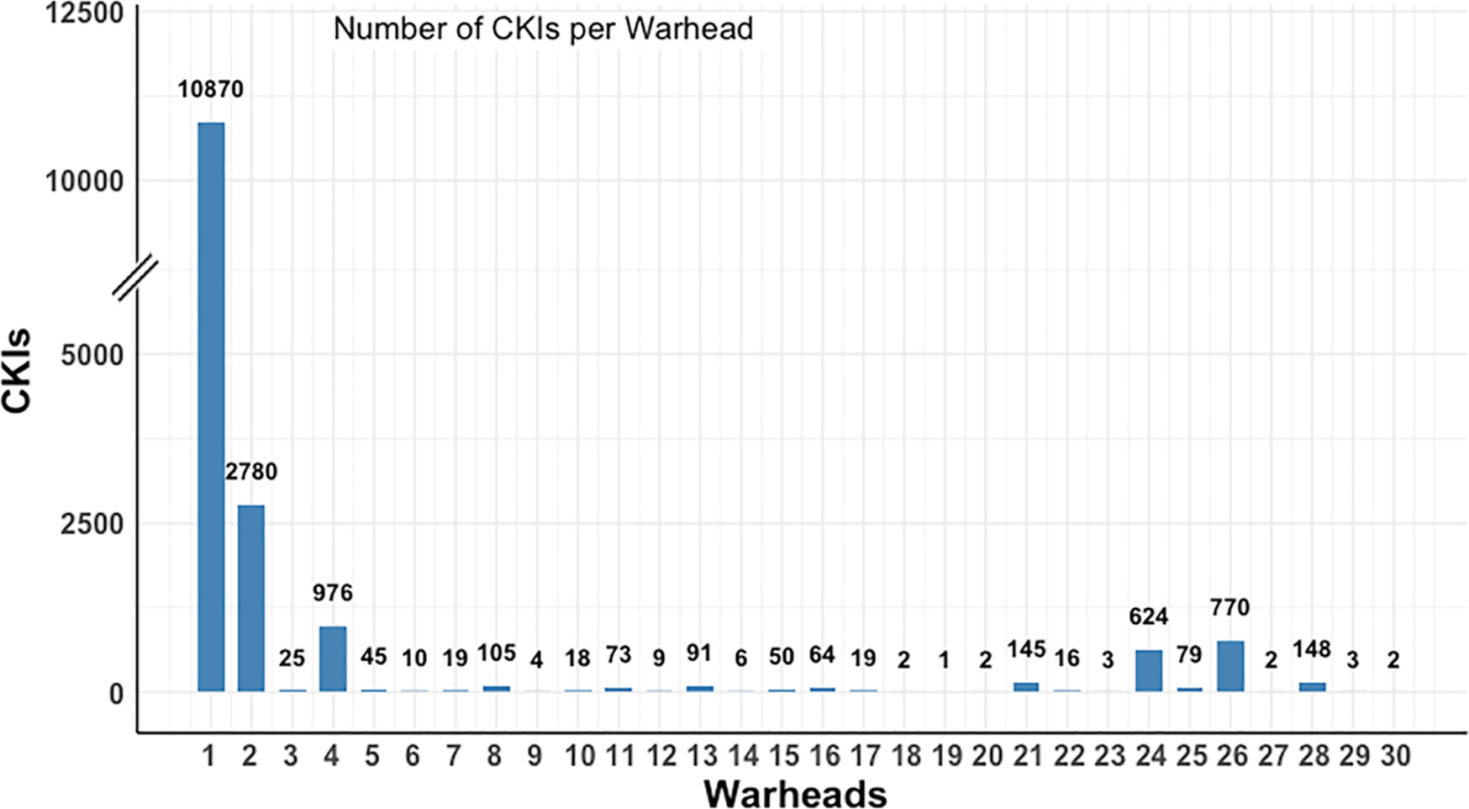
CKI warheads and the corresponding number of CKIs.

Dozens of warhead fragments have been successfully applied to the design of CKIs. Here, we surveyed recently published warhead-related reviews and selected 30 commonly used warheads for determining potential CKIs (Figure 4 and Method section).^2,18,22−26,29^ Notably, warheads **1**–**22** are often used to design irreversible CKIs while warheads **23**–**30** occur in RCKIs. Using the warheads as bait, the corresponding inhibitors equipped with these warheads were fished out and referred to as potential CKIs in our data set. Figure 5 shows the warheads and distribution of corresponding CKIs. Acrylamide and its derivatives account for the vast majority of CKIs with 10,870 (warhead **1**) and 2780 (warhead **2**) CKIs. The second primary component is butynamide and its analogue with 976 (warhead **4**) and 45 (warhead **5**) compounds. From the reversible warheads **23**–**30**, aldehyde and cyanoacrylamide dominate the distribution with 770 (warhead **26**) and 716 (warheads **23**–**25**) CKIs, respectively.

**Figure 6:**
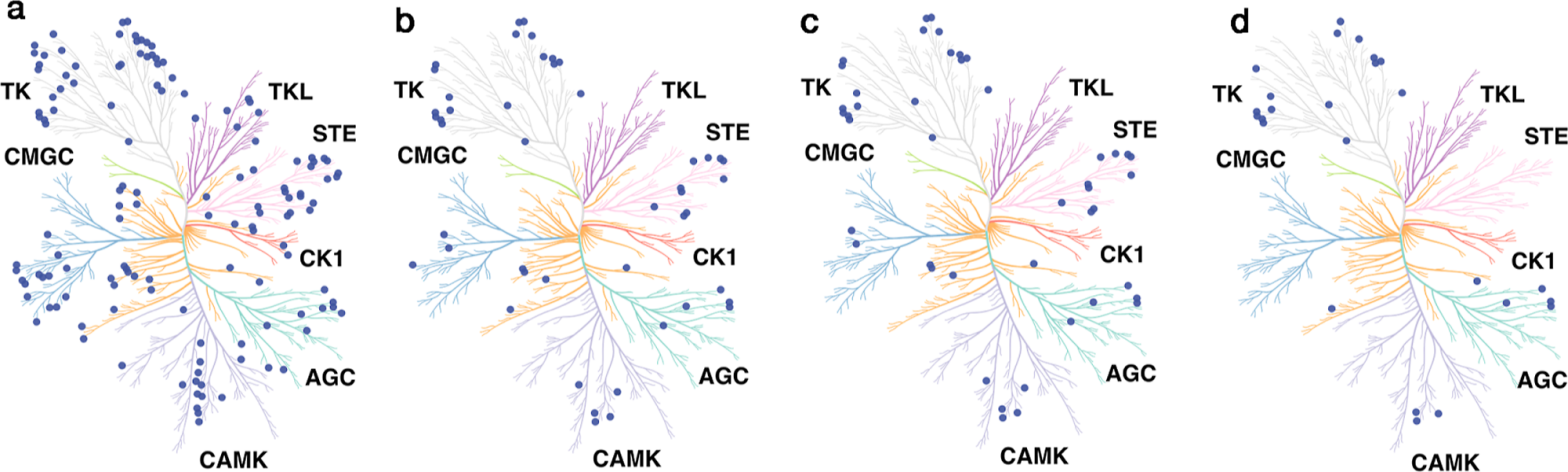
Distributions of warheads across the human kinome (dark blue balls). (a) Acrylamide-containing CKIs cover 132 kinases. (b) Butynamide-containing CKIs cover 43 kinases. (c) Aldehyde-containing CKIs cover 41 kinases. (d) Cyanoacrylamide-containing CKIs cover 21 kinases. Specific kinase names are found in the Supporting Information (Table S1).

CKIs with these warheads target different kinases (Figure 6). The 13,650 acrylamide-containing CKIs target 132 kinases (Figure 6a). The butynamide-containing CKIs target 43 kinases (Figure 6b). The aldehyde-containing CKIs target 41 kinases, and the cyanoacrylamide-containing CKIs target 21 kinases (Figure 6c,d). These warheads, which target multiple kinases, show privileged properties^22^ and provide a design strategy for repurposing these warheads in the development of new CKIs.^30^ It is worth noting that acrylamide, cyanoacrylamide, and butynamide are similar in structure because they all contain a common amide feature and have proved tractable in kinase covalent drug design. However, aldehydes have not been commonly used in drug discovery because they can undergo unexpected reactions with off-target enzymes, resulting in toxic side products.^31^ For kinase-targeted covalent drug design, an aldehyde that is used as a reversible warhead must be analyzed for its high reactivity and metabolic and chemical instability.^32−34^

### 2.3 Extended Warheads

**Figure 7:**
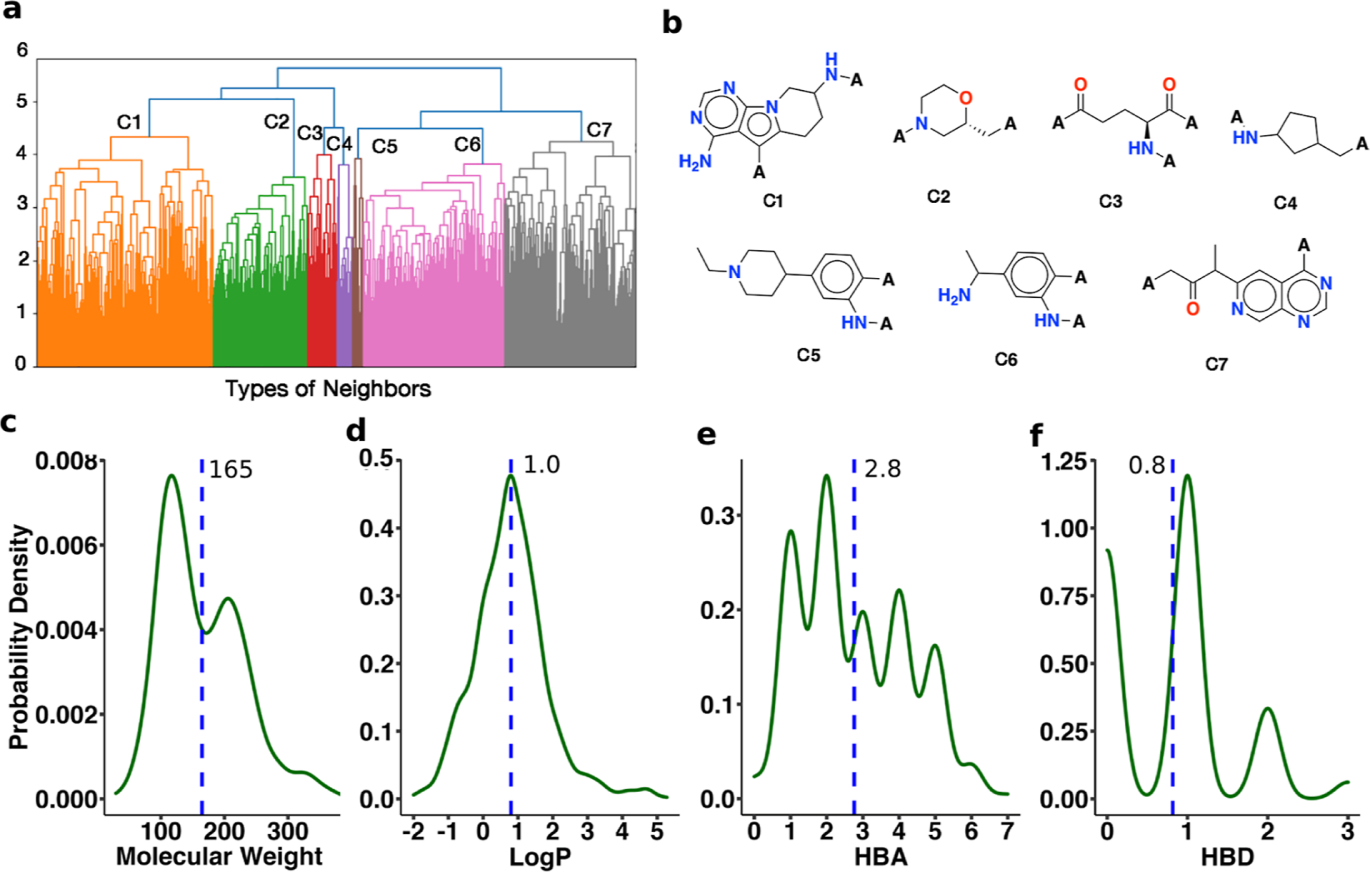
Hierarchical clustering. (a) 7 clusters of adjacent fragments are highlighted in different colors. (b) An example from every cluster illustrates the different types of adjacent fragments. (c–f) Properties of adjacent fragments: molecular weight (MW), log 10 value of *P* (log *P*, negative value for hydrophilic compounds), number of H-bond acceptors (HBAs), number of H-bond donors (HBDs), respectively. The *y*-axis shows the probability density. The dashed lines indicate the mean value and the corresponding abscissa.

Improving the selectivity and affinity of compounds by modifying hit-to-lead molecules is a key process in drug design. For the CKI design, it is important to have one electrophilic warhead to target the desirable nucleophile. Due to the limited choice of electrophile, modifying adjacent fragments of the warhead is necessary. Thus, we systematically analyzed the warheads and their adjacent fragments (together called extended warheads) to provide structural insights for designing CKIs. A total of 17,309 adjacent fragments with 1344 unique fragments comprised our adjacent fragment library (AFL, Table S2), bridging the scaffold with the corresponding warhead. Using hierarchical clustering analysis, adjacent fragments were clustered into 7 clusters (**C1–C7**, Figure 7a). Every cluster has different features, but 6 of the 7 are cyclo-based fragments (Figure 7b), such as aromatic ring-based (**C1**, **C5**, **C6**, and **C7**), heterocycle-based (**C2** and **C5**), or naphthene-based (**C4**). The adenine-derivative fragments (**C1** and **C7**) frequently interact with the hinge region like ATP.^12^ The molecular weight (MW) ranges from 29 to 388 g·mol^–1^ with the maximum density at 114.6 g·mol^–1^, the log *P* ranges from −2 to 5.28 with the maximum density at 0.8, the number of HBAs ranges from 0 to 7 with an average of 2, and the number of HBDs ranges from 0 to 3 with the maximum density at 1 (Figure 7c–f). These different properties illustrate the diversity of adjacent fragments, providing a broad conformational space for designing potential CKIs (Table S2).

**Figure 8:**
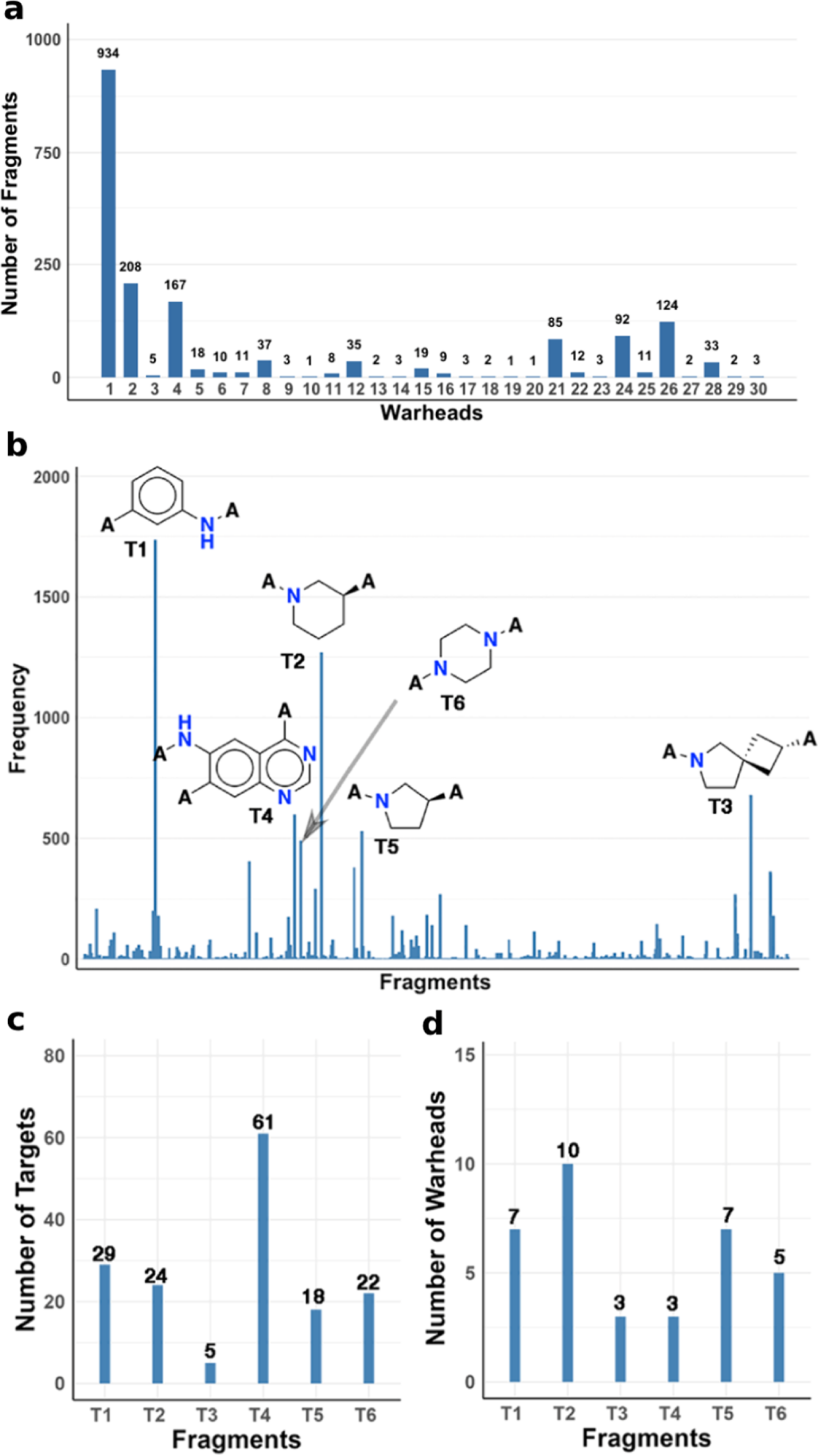
(a) Distribution of different fragments combined with different warheads. (b) Top 6 frequently used fragments out of 1344 unique adjacent fragments. (c) Number of kinases covered by the top 6 frequently used adjacent fragments. (d) Number of warheads equipped with the top 6 frequently used adjacent fragments.

We counted the frequency of occurrence of the 1344 different adjacent fragments in the CKI data set. Notably, 934 fragments occur in combination with warhead acrylamide (**1**), 208 occur in combination with warhead (**2**), 167 occur in combination with warhead (**4**), and 124 occur in combination with warhead (**26**) (Figure 8a). Among them, nearly 900 molecular fragments appear exclusively in 1 to 2 CKIs (Figure 8b). In contrast, there are 6 frequently used adjacent fragments: benzenamine (**T1**), piperidine (**T2**), 6-azaspiro[3.4]octane (**T3**), quinazoline (**T4**), pyrrolidine (**T5**), and piperazine (**T6**) (Figure 8b) found in >490 CKIs. They all occur in multiple CKIs targeting different kinases (Figure 8c) or are equipped with different warheads (Figure 8d), showing strong plasticity (adaptable to different CKI warheads or scaffolds). For example, **T2**, the adjacent fragment to 10 kinds of warheads, occurs in 24 kinases. The aromatic ring-based **T4**, as the adjacent fragment of 3 kinds of warheads, occurs in 61 kinases, showing strong resilience to different CKIs targeting different kinases. Of course, a variety of adjacent fragments yield different chemical properties, such as different structural sizes or being based on aromatic rings, offering a rich arsenal with versatile features for CKI design (Table S2).

### 2.4 Extended Warheads in Kinase-CKI Complexes

**Figure 9:**
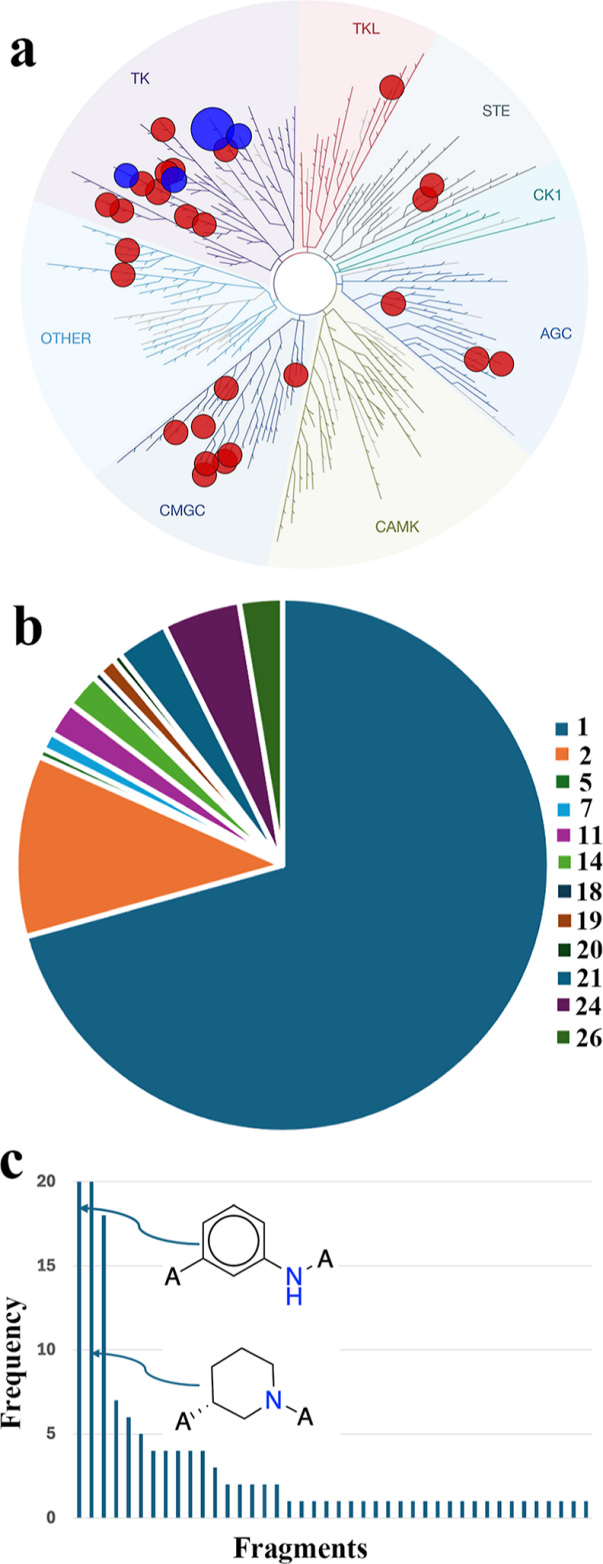
(a) Kinase-CKI complexes cover 30 different kinases, with the top 4 kinases highlighted in blue. (b) 12 types of warheads and their popularity. (c) 42 extended fragment of warhead 1. The top 2 2D structures are shown.

Based on our protocols for kinase-CKI complex data retrieval and curation (see Methods section), 190 kinase-CKI complexes from the CovalentInDB database and 1 from manual inspection of the latest updated KLIFS were obtained, respectively. Thus, a total of 191 PDB complexes were used as our kinase-CKI data set in the paper (see the full list in Supporting Information Table S3). These complexes were reported from 30 different kinases (Figure 9a), spanning various kinase families: TK, TKL, STE, AGC, and CMGC. Correspondingly, each kinase had a different number of kinase-CKI complexes, with EGFR having the highest number, totaling 58 kinase-CKI structures. Aside from EGFR, the kinases fibroblast growth factor receptor 4 (FGFR4), BTK, and JAK3 rank second to fourth in terms of the number of kinase-CKI complexes, which is consistent with the kinases with the Top4 number of CKIs (Figure 3).

There are 12 warheads (**1**, **2**, **5**, **7**, **11**, **14**, **18**, **19**, **20**, **21**, **24**, and **26** in Figure 4 and Table S3) that were used to form covalent interactions in the 191 complexes. Among them, warhead **1** (acrylamide) was the most common, appearing in 135 complexes, which accounted for approximately 71% (Figure 9b). The popularity of warhead **1** is in accordance with that in the CKI database (Figure 5). Moreover, we analyzed the extended fragments of warhead **1**, and 42 and different extended fragments were extracted (Table S4). These popular fragments were consistent with the top frequently used adjacent fragments from the CKI database among the extended fragments of warhead **1** (Figure 9c and Table S4). The extended fragments **T1** and **T2** are also the top 2 frequently used fragments in the CKI database (Figure 8b).

**Figure 10:**
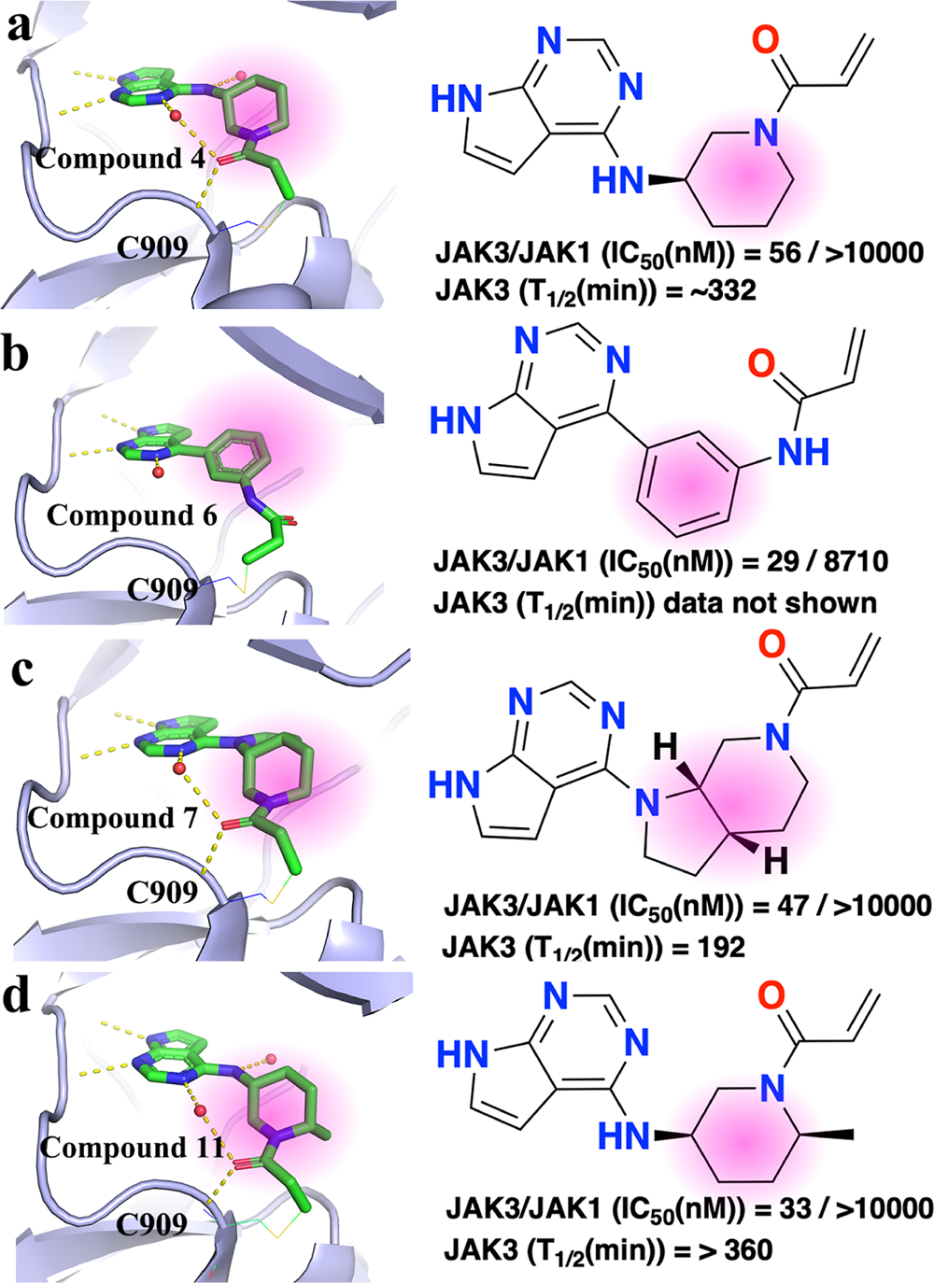
Binding modes, binding affinities, and pharmacokinetics of Compounds **4**, **6**, **7**, and **11** with extended fragments highlighted in pink. (a–d) PDB ids 5tts, 5ttv, 5ttu, and 5toz, respectively.

We further investigated the role of different extended fragments by analyzing the kinase-CKI complex database. Herein, we filtered out four crystallized structures that differ only in the extended fragment but have the same warhead 1 and core structures (Figure 10). The four structures were reported during the design of JAK3 kinase inhibitors for treating inflammatory diseases.^35^ The binding modes of the inhibitors are highly similar: covalently interacting with the same cysteine (Cys909), forming two H-bond interactions with the hinge region and interacting with one or two water molecules in the binding site (Figure 10a-d). However, subtle differences are still able to be observed. Compounds **4** and **11** both interact with two water molecules, respectively. In contrast, Compounds **6** and **7** each interact with only one water molecule. Moreover, the warhead acrylamide is observed making H-bond interactions with the NH of the Cys909 amide for compounds **4**, **7**, and **11**, respectively, but not for compound **6**. These nuances are also reflected in the differing binding affinities of JAK3 and JAK1 (IC_50_) and different pharmacokinetics (**T**_1/2_, Figure 10). As such, these different extended fragments likely play important roles in rational drug design.

### 2.5 Extended Warheads for RCKIs

**Figure 11:**
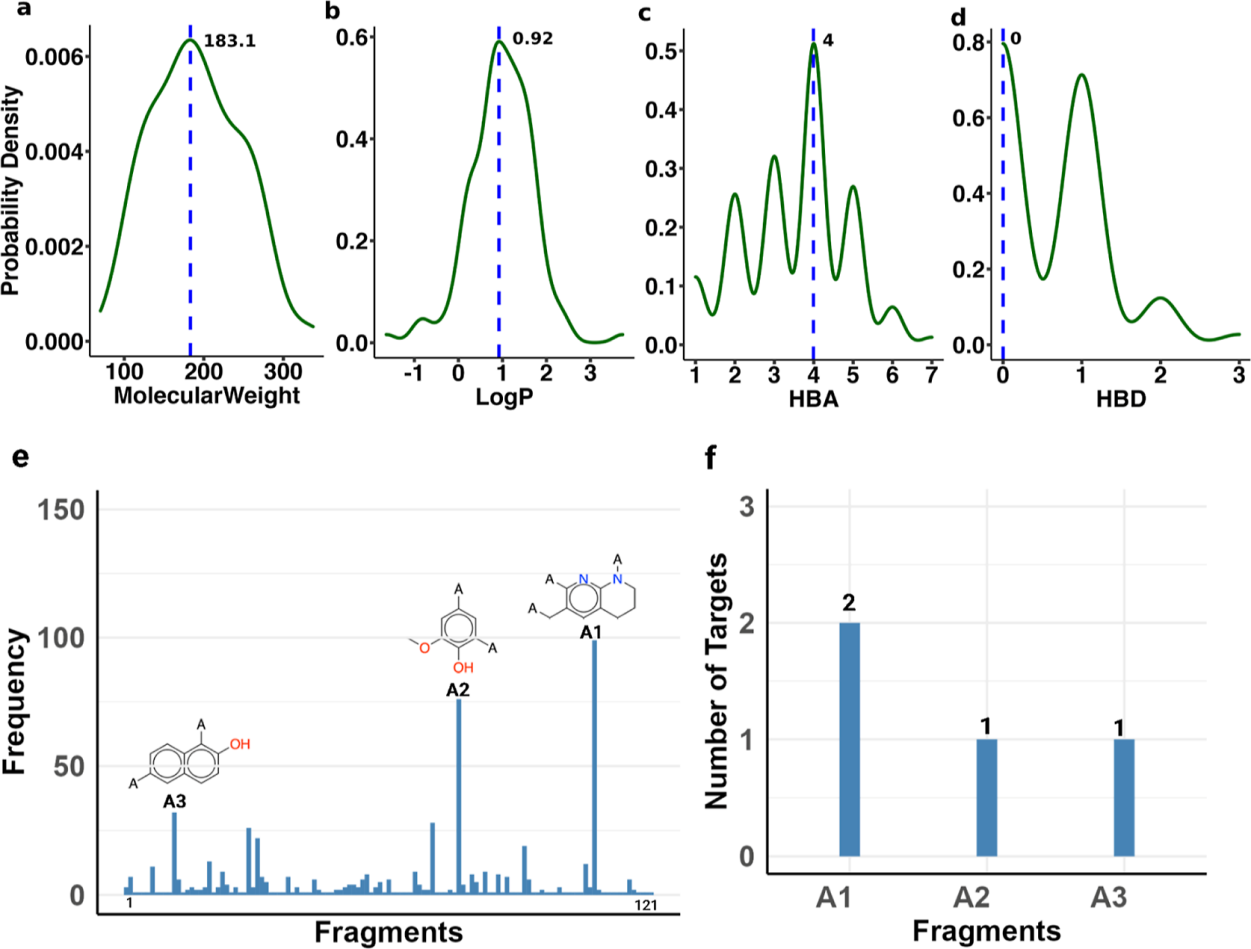
Adjacent fragments in aldehyde-equipped RCKIs. (a–d) Properties of adjacent aldehyde-equipped fragments. (e) Top 3 applied adjacent fragments. (f) The extended warhead targeting the number of kinases.

A concern with covalent inhibitors is unexpected toxicity due to off-target covalent modifications. RCKIs provide long-residence-time covalent binding but avoid permanent protein modification. Thus far, RCKIs have been established by tuning the warhead-nucleophile reactivity for ten kinases, such as EGFR, BTK, JAK3, and ribosomal S6 kinase 2.^36−40^ Correspondingly, multiple warheads have been used successfully in RCKI design, for example, warheads **23**–**30** (Figure 4). Given our CKI data, the largest number of RCKIs is 770 aldehyde-equipped CKIs (Figure 5, warhead **26**). Of the 770 RCKIs, there are 121 different adjacent fragments (Figure 11). The MW ranges from 70.1 to 337.4 g·mol^–1^ with the maximum density at 183.1 g·mol^–1^, log *P* ranges from −1.64 to 3.73 with the maximum density at 0.92, the number of HBAs ranges from 1 to 7 with an average of 4, and the number of HBDs ranges from 0 to 3 with the maximum density at 0 (Figure 11a–d). The top 3 are a tetrahydronaphthyridine fragment (**A1**), a methoxyphenol fragment (**A2**), and a betanaphthol fragment (**A3**), respectively (Figure 11e). All of the compounds have aromatic rings. The extended warheads target 1 to 2 kinases (Figure 11f). Currently, one of the RCKIs, Roblitinib (FGF401), which targets FGFR4 is used to treat hepatocellular carcinoma and other solid tumors with positive FGFR4 and klotho beta expression and is in a phase-II clinical trial.^34^

**Figure 12:**
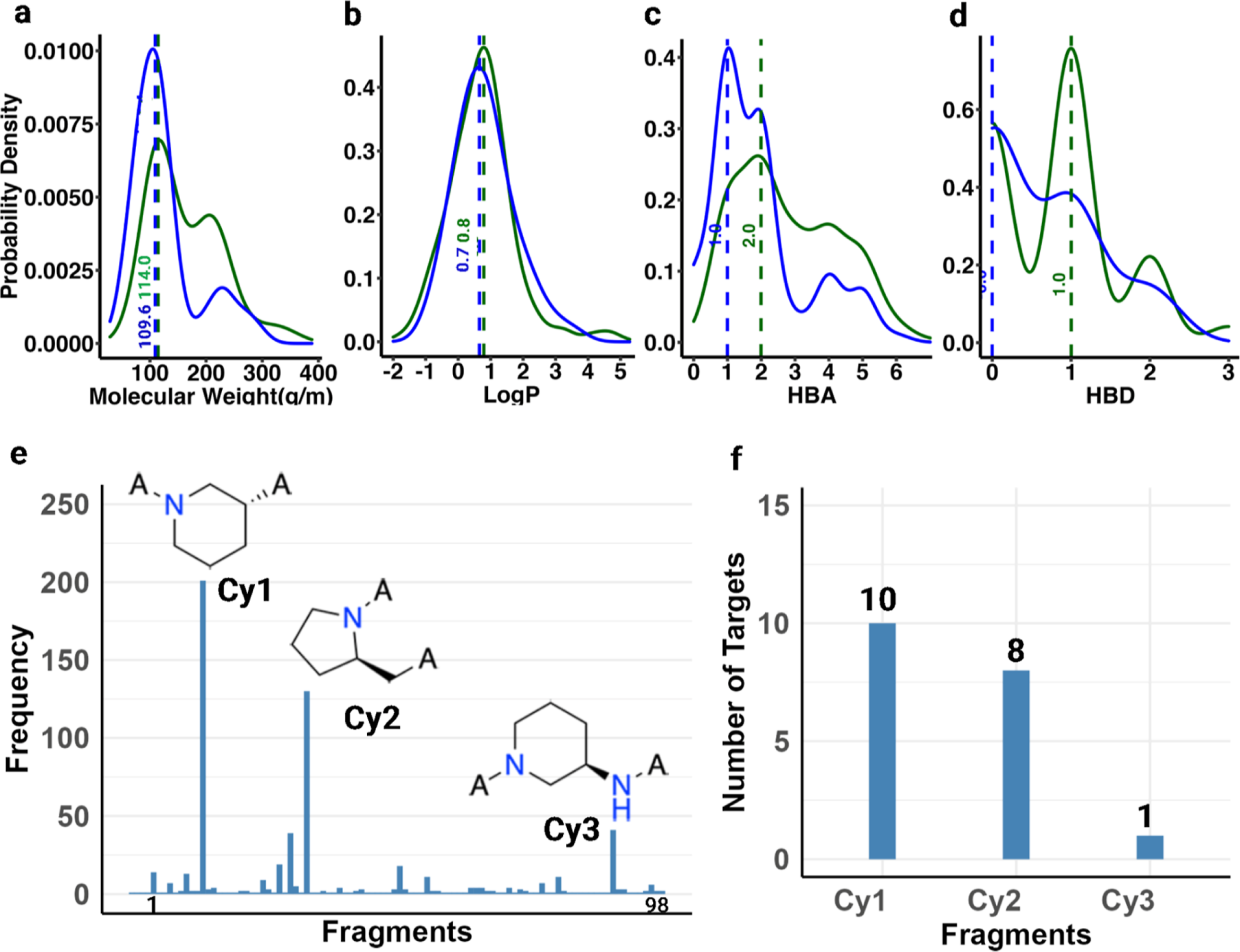
(a–d) Properties of adjacent acrylamide (in dark green) and cyanoacrylamide (in blue) equipped fragments in RCKIs, respectively. (e) Top 3 applied adjacent cyanoacrylamide-equipped fragments and (f) the corresponding extended warheads targeting the number of kinases.

Cyanoacrylamide-equipped CKIs are the second largest group, with 716 RCKIs from which 98 different adjacent fragments were extracted. The MW ranges from 43.1 to 297.4 g·mol^–1^ with the maximum density at 109.6 g·mol^–1^, log *P* ranges from −0.91 to 3.62 with the maximum density at 0.7, the number of HBAs range from 0 to 7 with an average of 1, and the number of HBDs range from 0 to 3 with the maximum density at 0 (Figure 11a–d). The adjacent fragment in acrylamide-equipped CKIs shows trends in MW and logP similar to those of the adjacent fragments in cyanoacrylamide-equipped CKIs. However, the number of HBAs and HBDs has increased slightly from 1 to 2 for HBAs, and from 0 to 1 for HBDs, respectively. The top 3 are piperidine (**Cy1**), 2-methylpyrrolidine (**Cy2**), and 3-aminopiperidine (**Cy3**) (Figure 12e). Compared to the aldehyde-based RCKIs, cyanoacrylamide-equipped RCKIs can adapt to targeting multiple kinases such as **Cy1** + cyanoacrylamide-based RCKIs targeting 10 kinases (Figure 12f). The top 1–3 adjacent fragments are all heterocycle-based (Figure 12e), different from the aromatic ring-base adjacent fragment of the aldehyde warhead (Figure 11e). It is worth noting that cyanoacrylamide is also a derivative of acrylamide. Currently, two cyanoacrylamide-equipped RCKIs, PRN473 and rilzabrutinib, are in phase-I and phase-III clinical trials, for treating pemphigus vulgaris and immune thrombocytopenia, respectively.^41,42^

## 3 Conclusions

Covalent drug design mainly focuses on designing an electrophile as a warhead to strike the accessible residues based on existing noncovalent inhibitors as scaffolds.^18^ Due to the limited types of warheads, additional adjacent fragments need to be considered in striking the desired residues. Here, we systematically explored adjacent fragments and the resultant warhead patterns, providing more opportunities to achieve covalent inhibition.

Systematic analysis of our current CKI data set found 16,961 covalent inhibitors covering 146 kinases, accounting for 70% of the 209 kinases that have at least one cysteine in the binding site. Further, we conducted systematic analysis of extended warheads, whereupon 1344 unique adjacent fragments associated with the corresponding warheads were extracted. The complete adjacent fragment library with associated properties can be accessed (Table S2) in which **T1–T6** shows the highest adaptability. Adjacent fragments increase the conformational space of the warhead, providing more options for refining the hit-to-lead molecules anchoring the desired nucleophile or even dual-nucleophiles.^43,44^

Along with the development of CKIs, more attention has been placed on RCKIs for exploiting sustained potency while avoiding unintended permanent protein modification.^45^ However, RCKI warheads are difficult to design. To help, we collated current RCKI warheads and extracted the corresponding adjacent fragments, with aldehyde and cyanoacrylamide showing broad utility. Importantly, their adjacent fragments present distinctive characteristics: aromatic ring-like adjacent fragments for aldehyde warheads; and heterocyclic-like ones for cyanoacrylamide warheads.

The diversity of adjacent fragments illustrates the potential for designing precise covalent inhibitors. Exploration of adjacent fragments and the corresponding warheads offers insights into the interaction modes of fragments, important for drug design using fragment blocks.^46^ The exploration of adjacent fragments described here should provide useful information as an arsenal for developing new CKIs with the appropriate warheads.

## 4 Methods

### 4.1 Curating the CKI Data Set

First, a CKI data set was curated and filtered for the purpose of extending warheads from different databases (accessed on 17 October 2023) including BindingDB,^47^ CheMBL,^48^ and an in-house CKI library including 200 manually curated CKIs (see reference *Pharmaceuticals* (*Basel*) **2022**, 15 (11)).^22^ A total of 521,736 kinase-compound pairs were obtained using thresholds of inhibitory activity values less than 10,000 nM (*K*_i_, *K*_d_, or IC50) and the highest confidence score of 9. Second, we double-checked that there are 208 kinases with available cysteines near the binding sites among the whole human kinome (see Table S5, which was adapted from the Supporting Information S3 in Gray’s papers).^2,18^ Third, we filtered all the kinase-compound data points that target the 208 kinases, resulting in 162,165 unique kinase-compound pairs. Concurrently, we reviewed recently published review papers to determine the warheads that have been commonly applied.^22,25,26,49^ As a result, we obtained 30 warheads that are considered common containing 22 irreversible and 8 reversible types of warheads.^30,40,50^ Finally, based on 30 warheads (Figure 4) that have been applied in practice, we extracted all the CKIs that have the warhead fragment. Duplicate kinase-CKI pairs were deleted regardless of the essay’s values from different sources. All CKIs were represented in a canonical SMILES format.

### 4.2 Kinase-CKI Data Set

A comprehensive list of crystallized kinase-CKI structures and their conformational data was compiled by integrating multiple sources: the CovalentInDB covalent inhibitor database,^27^ the KLIFS structural kinase database,^51^ UniProtKB/Swiss-Prot’s PDB cross-references,^52^ and kinase–ligand complex structures from the PDB.^53^ First, the CovalentInDB data set, updated on June 20, 2024, was downloaded. Second, a list of crystallized kinase structures from KLIFS (accessed August 22, 2024) was retrieved. Using this list, we filtered the CovalentInDB database to identify the PDBid list of kinase-CKI structures with a nucleophilic cysteine. Between June 20 and August 22, 2024, additional CKIs were manually verified from the KLIFS web site for completeness. Finally, the kinase-CKI 3D structures were obtained from the PDB database using the corresponding UniProt entries.^52^

### 4.3 Extracting Adjacent Fragments

Adjacent fragments were extracted using the Chem.Recap module of the RDKit chem lib.^54,55^ First, every compound was fragmented using the Recap method, which is a hierarchy of nodes. Then, we teased out those nodes with two leaves at the same branch, where if one is a warhead, the other is an adjacent fragment. Further, adjacent fragments at the distal end of CKIs are excluded, while those bridging warheads and scaffolds are retained. We traversed all CKIs to provide an adjacent fragment library (a framework module in Python is enclosed in Table S6). The warhead input format is represented as a SMART pattern using the module MolFragmentToSmarts in RDKit.^54^

Adjacent fragments were clustered using hierarchical clustering with an average linkage method based on pairwise cosine similarities of Morgan fingerprints (radius = 2, nBits = 2048) using RDKit.^54^ The properties of adjacent fragments were extracted using the Descriptors module in RDKit.^54^ All fragments were drawn using ChemDraw ṽv20.0.

## Data Availability

The compounds’ data in the paper were downloaded from the CheMBL at https://www.ebi.ac.uk/chembl and BindingDB at https://www.bindingdb.org. The extended warhead is extracted using in-house Python code that is released in the Supporting Information. The derived extended warhead library is published with the paper.

## Supporting Information Available

(PDF)
Table S1, Specific kinases covered by acrylamide-, butynamide-, aldehyde-, and cyanoacrylamide-equipped CKIs, respectively; Table S2, the complete adjacent fragment library; Table S3, kinase-CKI dataset; Table S4, the extended fragments of warhead acrylamide in the kinase-CKI dataset; Table S5, 208 kinases with available cysteines in the binding sites (XLSX)
Table S6, The Python framework for extracting adjacent fragments of CKIs (PDF)
