# Supplementary material for: Exploring Extended Warheads toward Developing Cysteine-Targeted Covalent Kinase Inhibitors
Source: J Chem Inf Model. 2024 Dec 10;64(24):9517–27. doi: 10.1021/acs.jcim.4c00890 (PMC11684028; doi:10.1021/acs.jcim.4c00890)
Supplement: Supplementary file 1 — ci4c00890_si_001.pdf [file ci4c00890_si_001.pdf]

## Supporting Information:

# Exploring Extended Warheads Toward Developing Cysteine-Targeted Covalent Kinase Inhibitors

Zheng Zhao* and Philip E. Bourne*

School of Data Science and Department of Biomedical Engineering, University of Virginia, Charlottesville, Virginia 22904, United States of America

*Corresponding author

Email: zz7r@virginia.edu (ZZ) and peb6a@virginia.edu (PEB)

Table **S1**: Kinases covered by acrylamide-, butynamide-, aldehyde-, and cyanoacrylamide-equipped CKIs, respectively (see the separate CSV file).

Table **S2**: Adjacent fragment library including the properties of molecular weight, LogP, Topological Polar Surface Area (TPSA), rotatable bonds, HBD, and HBA (see the separate CSV file).

Table **S3**: Kinase-CKI complex dataset.

Table **S4**: Extended fragments obtained from kinase-bound CKI complexes.

Table **S5**: 208 kinases with available cysteines in the binding sites (see the separate CSV file).

Table **S6**: Python framework for extracting adjacent fragments of CKIs.

```python
#given one CKI (named: mol) and the corresponding warhead (named: warhead)
#the neighbor and warhead were returned with the SMILES format

decomp = Recap.RecapDecompose(mol)
smiles_list = []
def get_neighbor(mol, warhead):
    if len(decomp.children.values()) == 2:
        for child in decomp.children.values():
            smiles_list.append(child.smiles)
        neighbor_and_warhead = ", ".join(smiles_list)
    elif len(decomp.children.values()) > 2:
        for child in decomp.children.values():
            if len(child.children) == 2:
                if child.mol.HasSubstructMatch(warhead):
                    for smiles_string in child.children:
                        neighbor_and_warhead = ", ".join(smiles_string)
            elif len(child.children) > 2:
                if child.mol.HasSubstructMatch(warhead):
                    get_neighbor(child, warhead)
    return neighbor_and_warhead
```
