# Supplementary material for: Exploring Extended Warheads toward Developing Cysteine-Targeted Covalent Kinase Inhibitors
Source: J Chem Inf Model. 2024 Dec 10;64(24):9517–27. doi: 10.1021/acs.jcim.4c00890 (PMC11684028; doi:10.1021/acs.jcim.4c00890)
Supplement: Supplementary file 3 — ci4c00890_si_009.pdf [file ci4c00890_si_009.pdf]

Table S6: Python framework for extracting adjacent fragments of CKIs.

<table>
<tr><th></th><th>Extracting adjacent fragments</th></tr>
<tr><td>Module (Python)</td><td><pre>
from rdkit import Chem
from rdkit.Chem import Recap

decomp = Recap.RecapDecompose(mol)
smiles_list = []

def get_neighbor(mol, warhead):
    if len(decomp.children.values()) == 2:
        for child in decomp.children.values():
            smiles_list.append(child.smiles)
        neighbor_and_warhead = ", ".join(smiles_list)
    elif len(decomp.children.values()) > 2:
        for child in decomp.children.values():
            if len(child.children) == 2:
                if child.mol.HasSubstructMatch(warhead):
                    for smiles_string in child.children:
                        neighbor_and_warhead = ", ".join(smiles_string)
            elif len(child.children) > 2:
                if child.mol.HasSubstructMatch(warhead):
                    get_neighbor(child, warhead)
    return neighbor_and_warhead
</pre></td></tr>
<tr><td>Usage</td><td>Input one CKI (named: mol) and the corresponding warhead (named: warhead), the warhead and its corresponding neighbors were returned with the SMILES format.</td></tr>
</table>
